# Epidemiological data on HIV-infected patients and the importance of education regarding the infection rate. An analytical cross-sectional study

**DOI:** 10.1590/1516-3180.2021.0368.R1.23072021

**Published:** 2022-02-07

**Authors:** Víctor de Oliveira Costa, Matheus Bresser, Bruna Malaquias Arguelles da Costa, Nathália Munck Machado, Marcos de Assis Moura

**Affiliations:** I MD. Physician, Internal Medicine, Faculdade de Ciências Médicas e da Saúde de Juiz de Fora (SUPREMA), Juiz de Fora (MG), Brazil; and Undergraduate Student, Physics, Universidade Federal de Juiz de Fora (UFJF), Juiz de Fora (MG), Brazil.; II Undergraduate Student, Medicine, Universidade Federal de Juiz de Fora (UFJF), Juiz de Fora (MG), Brazil.; III Undergraduate Student, Nursing, Faculdade de Ciências Médicas e da Saúde de Juiz de Fora (SUPREMA), Juiz de Fora (MG), Brazil.; IV PhD, Research Associate, Department of Population Health, University of Kansas Medical Center, Kansas City, Kansas, United States.; V MD, PhD. Professor, Internal Medicine, Faculdade de Ciências Médicas e da Saúde de Juiz de Fora (SUPREMA), Juiz de Fora (MG), Brazil; and Professor, Internal Medicine, Universidade Federal de Juiz de Fora (UFJF), Juiz de Fora (MG), Brazil.

**Keywords:** Acquired immunodeficiency syndrome, Education, Sexually transmitted diseases, Syphilis, Epidemiology, Academic degree, HIV-infected, Venereal diseases

## Abstract

**BACKGROUND:**

Sexually transmitted diseases (STIs) are an important public health problem in all countries. Knowledge of their relationship with the various socioeconomic levels is necessary for an understanding of their epidemiology and behavior in society.

**OBJECTIVE:**

To investigate the epidemiology of human immunodeficiency virus (HIV)-positive patients and to correlate education with history of sexually transmitted diseases, especially for syphilis.

**DESIGN AND SETTING:**

Analytical cross-sectional study carried out in the city of Juiz de Fora, Minas Gerais, Brazil.

**METHODS:**

The medical records of HIV/acquired immunodeficiency syndrome (AIDS) patients who started antiretroviral therapy (ART) between January 2010 and July 2018 were assessed. These patients were attended at the specialized assistance service for HIV/AIDS) of the Department of Sexually Transmitted Diseases (STD/AIDS) of the city of Juiz de Fora. In total, 335 patients were selected.

**RESULTS:**

In our sample, 73.13% were male; 57.36% were aged between 25 and 45 years and 24.23% were over 45 years of age. Regarding sexual orientation, 61.78% were homosexual. Regarding education, 52.88% had “unskilled education”, while 47.12% had “qualified education”. Analysis on the relationship between schooling and syphilis, a positive relationship between qualified schooling and syphilis was observed: odds ratio = 3.588; 95% confidence interval: 1.090-11.808.

**CONCLUSION:**

Homosexual male patients are most affected by HIV. Furthermore, this disease is not limited only to individuals with low education. Syphilis should be suspected in all individuals.

## INTRODUCTION

Sexually transmitted infections (STIs) are a public health problem and affect the lives of people around the world. This situation is associated with high rates of transmission, often explained by the view that people are not well informed about the transmission of these diseases or ignore the mandatory precautionary measures for safe sex. Individuals infected with any STI are five to ten times more likely than non-infected individuals to acquire or transmit the human immunodeficiency virus (HIV) through sexual contact.^1^ Many asymptomatic or undiagnosed individuals transmit HIV, syphilis and hepatitis B and C, either sexually or through contact with contaminated blood, as in the transmission of hepatitis C.^2^

In Brazil and worldwide, HIV infection persists and is often associated with other STIs. Co-infection with HIV and syphilis, for example, has synergistic action and increases the transmissibility of HIV.^3^ According to the Brazilian Ministry of Health, syphilis cases have increased mainly among people with “qualified” education level. This also represents a major problem and risk with regard to the transmissibility of HIV.^4^

## OBJECTIVE

The objectives of this study were to outline the epidemiological data on HIV-positive patients, and to correlate these patients’ education with occurrence of other STIs, such as syphilis, in Juiz de Fora, Minas Gerais.

## METHODS

### Design, ethical aspects and procedures

This was an analytical cross-sectional study. The medical records of HIV/acquired immunodeficiency syndrome (AIDS) patients who started ART between January 2010 and July 2018 were assessed. These patients were attended at the specialized assistance service (SAE) of the Department of Sexually Transmitted Diseases (STD/AIDS) of the city of Juiz de Fora. The survey took place between July 2019 and December 2019. The research was started after obtaining approval from the Research Ethics Committee of the Faculdade de Ciências Médicas e da Saúde de Juiz de Fora (SUPREMA), under opinion report number 2.722.176, dated June 19, 2018. The criteria of trust and privacy were guaranteed to the participants, in accordance with Resolution 466/2012 of the National Council for Research Ethics (CONEP), which deals with research involving human beings.

### Participants

The participants in this study were HIV/AIDS patients who were treated at the SAE. The inclusion criteria were that the patients needed to: 1) be attended at the SAE of the Department of Sexually Transmitted Diseases (STD/AIDS) of the city of Juiz de Fora; 2) be a carrier of the HIV/AIDS virus, as confirmed through laboratory tests. The exclusion criterion were that patients were not selected if the medical records were poorly written or had non-conformities that could generate confusion bias, such as illegible handwriting.

For this study, the medical records were separated into three groups: group A, patients who started ART with dolutegravir (DTG), with immediate adherence; group B, patients who started ART without DTG, with adherence in 45 days; and group C, patients who started ART without scheduled adherence and with different treatment schedules. In the end, the study sample comprised 335 participants.

### Data analysis

The information provided was transcribed and tabulated using the Windows Excel software 2013 (Microsoft Corporation, Redmond, Washington, United States). The data on these spreadsheets was then transferred to the Statistical Package for the Social Sciences (SPSS) software, version 23.0 (released 2015) (IBM Corp., Armonk, New York, United States), in which a statistical analysis was performed. The mean values and standard deviations for numerical variables were calculated. The statistical significance level established was P < 0.05.

### Adjusted analysis

We constructed a logistic regression model to determine the interaction between schooling and sexually transmitted infections. First, statistical comparisons were made between schooling and other variables using the Wilcoxon signed-rank test for continuous data and Fisher’s exact test, two-sided for nominal variables. Then, the variables that were significantly correlated at P > 0.05 (age, sex and syphilis) were included in the logistic regression model. Adjusted odds ratios (OR) and 95% confidence intervals (95% CI) were calculated. The logistic regression was performed using the R Core Team software, version 4.0.2 (R Foundation for Statistical Computing, Vienna, Austria).

## RESULTS

This study evaluated 335 patients with HIV. The epidemiological characteristics of these patients are shown in Table 1.

**Table 1.** Epidemiological data on human immunodeficiency virus-positive patients

Sample epidemiology					Frequency	Percentage
**Sex**	Female				82	24.48%
	Male				245	73.13%
	Transgender				8	2.39%
**Total**					**335**	**100.00%**
**Age (years)**	Average				36.43	SD: 13.081
	Maximum				76	
	Minimum				14	
	< 25				60	18.40%
	25-45				187	57.36%
	> 45				79	24.23%
**Total**					326	100.00%
**Sexual orientation**		**F**	**M**	**T**		
	Bisexual	0%	5%	95%	18	8.00%
	Heterosexual	47%	0%	53%	68	30.22%
	Homosexual	94.3%	5%	0.7%	139	61.78%
**Total**					**225**	**100.00%**
**Partner serological status**	Unknown				106	60.23%
	HIV-				15	8.52%
	HIV+				55	31.25%
**Total**					**176**	**100.00%**
**Previous STIs**	No				44	50%
	Yes				44	50%
**Total**					**88**	**100%**
**Hepatitis B vaccination**	No				6	1.79%
	Yes				1	0.30%
	Without knowledge				328	97.91%
**Total**					**335**	**100.00%**
**Education**	Complete elementary education				31	11.15%
	Incomplete elementary school				72	25.90%
	Complete high school				70	25.18%
	Incomplete high school				44	15.83%
	Complete higher education				30	10.79%
	Incomplete higher education				31	11.15%
**Total**					**278**	**100.00%**

SD = standard deviation; STIs = sexually transmitted infections; F = female; M = male; T = transgender.

Among these 335 HIV-positive patients, 82 (24.48%) were female, 245 (73.13%) were male and 8 (2.39%) were transgender. In terms of age, the sample was divided into three parts: less than 25 years, from 25 to 45 years and more than 45 years, with the purpose of separating the sample population into three age ranges. There were 60 patients (18.40%) aged less than or equal to 24 years; 187 patients (57.36%) aged between 25 and 45 years and 79 patients (24.23%) over 45 years. The maximum age was 76 years, the minimum was 14 and the average was 36.43 (standard deviation, SD: 13.081). There were nine participants whose age was not identified.

Regarding sexual orientation, 225 patients were evaluated. Among these, 18 patients claimed to be bisexual (8.0%), 68 patients claimed to be heterosexual (30.22%) and 139 patients claimed to be homosexual (61.78%).

**Figure 1.**
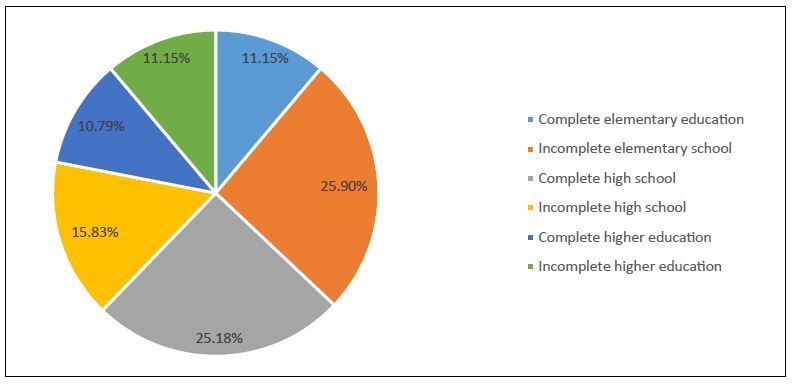
Graph representing the schooling of the sample studied.

Regarding educational level, 278 patients provided this information. Among these: 72 (25.9%) had been educated as far as incomplete elementary school; 31 (11.5%) as far as completed elementary school; 44 (15.83%) as far as incomplete high school; 70 (25.18%) as far as completed high school; 31 (11.15%) as far as incomplete higher education; and 30 (10.79%) as far as completed higher education. This information can be seen in the graph of Figure 1.

In the first visits, rapid tests for syphilis and hepatitis B and C serological tests were performed (Table 2). The test for syphilis was done on 95 patients, among whom 48 were reactive (50.53%); the test for hepatitis B virus (HBV) was done of 48 patients, with one reactive case (2.08%); and the test for hepatitis C virus (HCV) was done on 54 patients, among whom none were reactive.

**Table 2.** Rapid test for communicable diseases

Rapid test						
Result	Syphilis	Percentage	HBV	Percentage	HCV	Percentage
Reactive	48	50.53%	1	2.08%	0	0
Non-reactive	47	49.47%	47	97.92%	54	100.00%
**Total**	**95**	**100.00%**	**48**	**100.00%**	**54**	**100.00%**

HBV = hepatitis B virus; HCV = hepatitis C virus.

In order to investigate correlations between educational level and sexually transmitted infections, an odds ratio (OR) analysis was performed through logistic regression. The individuals analyzed were separated into two groups: the first was named “qualified education” and was composed of individuals who had reached the levels of completed high school or incomplete or completed higher education; the second was named “unskilled education” and was composed of individuals who had reached the levels of incomplete or completed elementary school or incomplete high school.

Given that skilled education is age and sex-dependent, a logistic regression analysis was performed, with adjustments for these variables (Table 3). The difference between qualified and unskilled education was found to be nonsignificant in this adjusted model (P = 0.675), as was age (P = 0.581). However, being male (OR 3.9; P = 0.055) and being positive for syphilis (3.588; P = 0.035) were significantly correlated with qualified education.

**Table 3.** Logistic regression results

Variable		Standard deviation	Odds ratio	P-value
**Schooling**	Qualified		Reference	0.675
	Unskilled	1.180	0.610 (0.060-6.164)	
**Age**		0.023	0.987 (0.943-1.033)	0.581
**Sex**	Female		Reference	0.055
	Male	0.719	3.961 (0.967-16.220)	
**Syphilis**	Non-reactive		Reference	0.035
	Reactive	0.607	3.588 (1.090-11.808)	

## DISCUSSION

Between 2007 and June 2019, 300,496 cases of HIV infection in Brazil were reported in the country’s notifiable diseases information system (SINAN), of which 136,902 cases (45.6%) were in the southeastern region. During this period, a total of 207,207 cases (69.0%) were reported among men and 93,220 cases (31.0%) among women. The sex ratio for the year 2018 was 2.6 (M:F), i.e. 26 men for every ten women.^5^ In the sample studied, a ratio of 2.98 (M:F) was observed, i.e. close to 30 men for every 10 women. We separated transgender individuals from these proportions, in order to ascertain the prevalence of this population in the sample.

Over the same period, regarding age groups, it was observed that most cases of HIV infection were in the range from 20 to 34 years of age, which accounted for 52.7% of the cases. Among the cases in which the educational level was informed, most of these individuals had completed high school, which represented 20.7% of the total. In another study, 12.1% of the cases had reached an incomplete schooling level between the 5^th^ and 8^th^ grades.^5^ In our sample, a similar number of individuals in this same age group was seen, and 57.36% of them were between 25 and 45 years old, thus showing higher prevalence in the generations of the 1980s and 1990s. In addition, 44.12% had completed high school, which shows that some patients with STIs now have qualified education levels, i.e. that STIs are not just prevalent among individuals with less school education.

In addition, among men, over the period observed, it was found in another study that 51.3% of the cases were due to homosexual or bisexual exposure, while 31.4% were heterosexual.^5^ In our sample, 61.78% of those infected were homosexuals, and 94.3% of them were male. Among women, it has been noted that 86.5% of the cases fall into the category of heterosexual exposure in Brazil.^5^ In the sample of our study, 53% of the heterosexuals were female and about 95% of the bisexuals were female. This situation demonstrates that the population most affected is still that of individuals with homosexual sexual orientation and is concentrated more in the male sex, while in the heterosexual group there is a tendency towards equality between the groups, i.e. in this group the female and male sexes have similar distributions.

This situation is similar to what has been seen in the United States. Homosexual and bisexual men together form a group that corresponded to 86% of new infections in the United States and its dependent territories in 2017. Homosexual exposure among men corresponded to 79% of the infections diagnosed that year, with or without an association with the use of injectable drugs, i.e. a proportion considerably higher than the 51.3% seen in Brazil.^6^ However, regarding American women, 76% became infected through heterosexual exposure and another group of 21%, through injecting drugs.^6^

HIV affects approximately 35 million people worldwide, and approximately 8.6% of them are co-infected with HBV.^7^ The rates of HIV/HBV co-infection vary according to the origin of the population studied and the geographical location.^8,9^ In a group of 297 patients evaluated at the Hospital de Clínicas, Universidade Federal do Paraná (UFPR), Brazil, the prevalence of hepatitis B markers was significantly associated with HIV infection, in comparison with the prevalence observed in the general population of the same geographical area.^10^ Out of the 48 patients analyzed in our study, only one had positive HBV serological tests. In addition, the prevalence of HIV/HCV co-infection was low in the group analyzed here, and this was also observed in studies in Recife and Pará.^11,12^ In a hospital in Porto Alegre, Brazil, anti-HCV was observed in 126 out of 330 cases (38.2%).^13^ This situation confirms the idea that the prevalence of these associations depends on the geographical location.

In 2018, 158,051 cases of acquired syphilis were reported to SINAN. This condition has been subject to compulsory notification since 2010. Its detection rate increased from 34.1 to 75.8 cases per 100,000 inhabitants between 2015 and 2018. In 2018, among the cases in which the education level was reported, 39.5% of these individuals had reached at least high school education.^4^

In the group analyzed here, a positive correlation with individuals with higher education levels was observed: these individuals were more likely to have contracted syphilis than individuals with lower education. The main limitation to this finding was the number of individuals from whom the rapid test results were notified (92 patients). Nonetheless, this relationship is extremely important from a public health point of view, given that it may indicate that campaigns to prevent these diseases are not being effective, since individuals with higher socioeducational levels do not follow preventive measures. It is worth mentioning that the population studied consisted of individuals with HIV and, therefore, presented higher risk of severe forms.

Education is associated with STIs, as observed in a previous survey conducted in São Paulo.^14^ An association between past STIs and lower levels of schooling has been described in the literature.^15^ However, because of the limitation of the size of the population studied, we were unable to add data from other STIs to the logistic regression analysis, such as hepatitis B and C.

## CONCLUSION

Our analysis shows that HIV is still a prevalent disease in society, and is more prevalent among males and homosexuals. It is similarly distributed across all educational levels; i.e. individuals with qualified education have a prevalence similar to that of individuals with unskilled education. This demonstrates that this public health problem transcends socioeducational levels.

In addition, syphilis is an emerging problem in the context of public health, and it should receive special attention for the entire population. Its incidence is not restricted mainly to less-favored socioeducational groups.
